# Spirulina platensis versus silymarin in the treatment of chronic hepatitis C virus infection. A pilot randomized, comparative clinical trial

**DOI:** 10.1186/1471-230X-12-32

**Published:** 2012-04-12

**Authors:** Mostafa Yakoot, Amel Salem

**Affiliations:** 1 Green Clinic and Research Centre, Alexandria 21121, Egypt; 2 Mabarrah Clinics, Alexandria, Egypt

**Keywords:** Chronic HCV, Spirulina platensis, Cyanobacteria, Silymarin, Safety and Efficacy

## Abstract

**Background:**

Spirulina platensis, a cynobacterium used frequently as a dietary supplement had been found to exhibit many immune-stimulating and antiviral activities. It had been found to activate macrophages, NK cells, T cells, B cells, and to stimulate the production of Interferon gamma (IFN-γ) and other cytokines. Natural substances isolated from Spirulina platensis had been found to be potent inhibitors against several enveloped viruses by blocking viral absorption/penetration and some replication stages of progeny viruses after penetration into cells. We aimed to study whether this dietary supplement possesses any therapeutically feasible activity worthy of further larger controlled clinical evaluation.

**Methods:**

Sixty six patients with chronic hepatitis C virus infection and eligible for inclusion had been randomized to either Spirulina or Silymarin treated groups for a period of six months treatment.

The two groups were followed up and blindly compared for early (after 3 months) and end of 6 months treatment virological response. The effects of both treatments on each of alanine aminotransferase (ALT), Chronic Liver Disease Questionnaire scores (CLDQ), Arizona Sexual Experience Scale scores (ASEX) and the occurrence of any attributable adverse events were also compared.

**Results:**

Among the 30 patients who had been treated with Spirulina and completed the 6 months protocol, 4 patients (13.3%) had a complete end of treatment virological response and 2 patients (6.7%) had a partial end of treatment response defined as significant decrease of virus load of at least 2-logs_10_. Though the proportion of responders in Spirulina group was greater than in the Silymarin group, the difference was not statistically significant at the end of both 6 months (p = 0.12) and 3 months treatment (p = 0.22) by Exact test. Alanine aminotransferase as well as CLDQ and ASEX scores were found to be more significantly improved in Spirulina than in Silymarin treated group.

**Conclusions:**

Our results could suggest a therapeutically feasible potential for Spirulina platensis in chronic HCV patients, worthy to conduct a larger sized and longer study to confirm these safety and efficacy encouraging results.

**Trial Registration:**

WHO Clinical Trial Registration ID: ACTRN12610000958088

http://apps.who.int/trialsearch/trial.aspx?trialid=ACTRN12610000958088

## Background

Infection with hepatitis C virus (HCV), an enveloped virus that belongs to the *Flaviviridae *family of positive-strand RNA viruses, is a major cause of chronic liver disease. WHO estimated that about 170 million people, (3% of the world's population), are infected with HCV and 3-4 million persons are newly infected each year [1-3].

Prevalence rates vary widely, ranging from 0.15% in Scandinavia to about 15% in Egypt [4]. The overall prevalence in the United States is 1.8%, corresponding to an estimated 3.9 million persons with HCV infection [5].

About 80% of newly infected patients progress to develop chronic infection. Cirrhosis develops in about 10% to 20% of persons with chronic infection. Liver cancer develops in 1% to 5% of persons with chronic infection over a period of 20-30 years [5,6].

Apart from its numerous contraindications and severe adverse effects, the current recommended treatment for chronic hepatitis C is not only too costly for most persons in developing countries to afford but also weakly effective [6].

A meta-analysis showed that only about 17% of patients with chronic hepatitis C obtained a sustained virological response on interferon monotherapy, which was the only recommended treatment until the late 1990 [7].

The current gold standard therapy is a combination of peg-interferon alfa and ribavirin [8,9]. Patients with genotype 1 infection have a 42%-51% likelihood of achieving a sustained virological response (SVR) after 48 wk of therapy. For patients with genotype 4 (approximately 90% of patients with hepatitis C in Egypt), the sustained virological response (SVR) rate with 48 weeks of therapy ranges from 50% to 60%. A significant proportion of treated patients thus either fail to respond or relapse following an initial response, and a substantial number of patients are unable to tolerate treatment [10].

Though around 78%-82% of patients with genotype 2 or 3 infection respond to 24 wk of treatment, those with high viral load are difficult to treat (< 70% responders) [11].

Generally, non-responders to prior standard bitherapy respond to retreatment in about 13% of the cases and relapsers in 58.5% of the cases [12].

Therapy requires weekly subcutaneous injections, twice-daily oral dosing and frequent visits, with blood tests. Side effects occur in nearly all patients. As a result, 15%-20% of patients in clinical trials and > 25% in clinical practice discontinue therapy.

Ribavirin does not appear to be effective when used alone. Furthermore, there is no clear evidence as to whether treatment reduces the risk of liver related morbidity or mortality [13].

An alternative treatment which is less costly, safer and more efficacious is being the search of many research authorities al-over the world.

Many herbal products had been studied and proved to offer some hepatoprotective activity and showed some benefits in the treatment of viral hepatitis, Glycyrrhizin, Silymarin, Curcumin, Schiszandra are examples [14-18].

Spirulina is a blue-green alga (cyanobacterium) that has been consumed as food in many countries since ancient times. It is presently marketed as a food supplement (nutraceutical) due to its high contents of proteins, γ-linolenic acid, vitamins and minerals [19]. Many toxicological studies have proven Spirulina's safety. Spirulina now is listed by the US Food and Drug Administration under the category Generally Recognized as Safe (GRAS) [20-24].

Spirulina and many other Cyanobacteria had been found to exhibit many immune-stimulating and antiviral activities not only in-vitro but also in animals and human volunteers. It had been found to activate macrophages, NK cells, T cells, B cells, and to stimulate the production of antibodies and cytokines. It enhances Interferon gamma (IFN-γ) production in an interleukin12, 18 (IL-12/IL-18)-dependent fashion [25-29].

Many natural substances derived from blue-green algae such as Sulfated homopolysaccharides and Sulfoglycolipids demonstrated monocyte/macrophage activation properties and found to substantially increase mRNA levels of key cytokines like interleukin-1beta (IL-1beta) and tumor necrosis factor-alpha (TNF-alpha) [30].

Calcium spirulan (Ca-SP), a natural sulfated polysaccharide, isolated from Spirulina platensis had been found to be a potent inhibitor against several enveloped viruses. Ca-SP was shown to target not only viral absorption/penetration stages but also some replication stages of progeny viruses after penetration into cells [31,32].

Whereas Cyanovirin-N (CV-N)[33-35] and Microvirin (MVN)[36,37] are two examples of mannose specific lectins isolated from blue green algae. They had been found to potently inactivate diverse strains of HIV and other enveloped viruses in nanomolar concentrations through preventing essential interactions between the virus envelope glycoprotein and target cell receptors [33-35].

At low nanomolar concentrations, CV-N was demonstrated to bind to HCV envelope glycoproteins, to block the interaction between the envelope protein E2 and CD81, a cell surface molecule involved in HCV entry [38,39].

The availability of Spirulina as an over the counter safe dietary supplement with reasonable costs added to the abovementioned data supporting both antiviral and immune-stimulant activities urged us to conduct this pilot clinical trial to test safety and efficacy of this supplement in patients with chronic hepatitis C.

## Methods

### Study design and setting

The study was conducted in an outpatient setting according to a randomized, double-blind, comparative study design with mean follow-up of 6 months.

### Patients

#### Sample size

To detect a 0.17 difference in proportion of responders between both comparison groups, with 80% power and two tailed 5% level of significance, the minimal sample size was calculated to be 30 for each group.

Sixty six eligible patients with documented chronic hepatitis C, genotype 4 had been included in the study according to the following criteria:

#### Inclusion criteria

• Chronic hepatitis C infection genotype 4 with PCR positive test +/- elevated liver enzymes.

• Males or females between 18 and 70 years old.

• Interferon naive (not previously treated with interferon alone or combined with ribavirin therapy).

• Relapsers (patients with a transient virological response to previous interferon or combined therapy), or non-responders to interferon or combined therapy are eligible if they stopped the antiviral drugs at least 3 months before recruitment.

#### Exclusion criteria

• Pregnant females.

• Patients with other causes of hepatitis, concurrent HIV virus infection, or active Schistosomiasis.

• Critically ill complicated patients with severe hepatic failure, malignancy or multi-organ failure.

The study protocol was reviewed and approved by the local ethical committee according to the Declaration of Helsinki. All subjects gave written, informed consent before any study-related procedures were performed.

Eighty five consecutive patients presenting to the 2 outpatient clinics from October 2008 to March 2010 as known cases of chronic hepatitis C infection or just newly diagnosed from history, clinical examination, 3^rd ^generation ELISA plus/or minus elevated liver enzymes were assessed for eligibility, by being subjected to full history taking, clinical examination and the following tests:

• Detection of HCV-RNA by PCR quantitative measurements by COBAS Amplicor 2.0, Roche Molecular Diagnostics, Pleasanton, CA, USA (lower limit of detection of 50 IU/mL).

• Screening test for HBsAg, anti-HIV, Shistosoma antigens.

• Upper abdominal and liver ultrasonography,

• Liver and kidney functions tests, urine analysis, stools analysis, complete blood count.

Patients fulfilling the inclusion/exclusion criteria were randomly divided into 2 arms:

1. An experimental group who started treatment with Spirulina 500 mg dry powder extract capsules (a product of Beovita-Safe Pharma, a German - Egyptian Pharmaceutical Company, headquartered in Alexandria, Egypt) in a dose of one capsule 3 times daily for a period of 6 months.

2. A control group who took Silymarin 140 mg capsules (generic product prepared by the same company) in a dose of one capsule 3 times daily for the same period.

Both drugs were packed in similar containers of 60 capsules of similar color and shape, packed and coded by Beovita Safe Company researchers blinded to the randomization process.

Randomization was done using software generated block randomization technique and both patients and investigators were blinded for the allocated drug. The randomization list and the drug codes were locked in sealed envelopes till the end of follow up period, final assessment and statistical analysis.

#### Outcome measures

1. Complete virological response (defined as loss of detectable hepatitis C virus RNA at the end of 6 months treatment (c-ETR) and at the end of 3 months treatment (complete early virological response (c-EVR)).

2. Partial virological response (defined as reduction of virus load by a minimum 2-log_10_) at the end of 3 months (p-EVR) and 6 months (p-ETR).

3. Biochemical response (normalization or significant reduction of elevated alanine tranaminase (ALT) at the end of treatment.

4. Improvement of health related quality of life scoring using the Chronic Liver Disease Questionnaire (CLDQ) developed by Younossi ZM et al. [40,41] The CLDQ includes 29 items in the following domains: abdominal symptoms, fatigue, systemic symptoms, activity, emotional function and worry. The response of CLDQ results in 1 to 7 scales: ranging from "1 = all of the time (worst)" to "7 = none of the time (best)". We compared the change of the final CLDQ overall score from baseline. CLDQ overall score is calculated by dividing the total score by the total number of items (29) resulting in a 1 to 7 scale [40,41].

5. The change in The Arizona Sexual Experiences Scale (ASEX) scores before and after 6 months of treatment was compared between groups. ASEX is a 5-item rating scale that quantifies sex drive, arousal, penile erection/vaginal lubrication, ability to reach orgasm, and satisfaction from orgasm. Possible total scores range from 5 to 30, with lower scores reflecting enhanced sexual function and higher scores reflecting impaired sexual function [42].

6. Occurrence of adverse events and toxicity: All subjects were examined and interrogated at each follow up visit for the occurrence and nature of any adverse events. All such events were recorded in the patients' case report forms. Adverse events were assessed for causality as probably related, possibly related, unlikely to be related or not related to study medications.

All patients in both groups had been followed up in monthly visits with physical examination, assessment of CLDQ and ASEX scorings, hematological studies, liver and kidney function tests and abdominal ultra-sonograms. Quantitative HCV-RNA by PCR test was done for each patient before treatment and after 3 and 6 months.

### Statistical analysis

Data were analyzed using the computer software package SPSS version 12, SPSS Inc. 233 South Wacker Drive, 11th Floor, Chicago, IL. Comparisons between means of paired scale variables before and after treatment were done using paired sample *t *test. Comparisons of means of scale dependent variables between the independent treatment groups were done using one way ANOVA or Student *t *test for independent samples. Z test for proportions, or Fisher's Exact tests were used for analysis of proportions and categorical variables. Multivariate analysis of covariance (MANCOVA) using the treatment group as the fixed independent factor with the baseline Child-Pugh score and the pre-treatment virus load as covariates to adjust for any initial group differences on the dependent outcome variables (the difference between baseline and post-treatment CLDQ and ASEX scores and their interaction). Statistical significance is considered when two sided p < 0.05.

## Results

**Figure 1:**
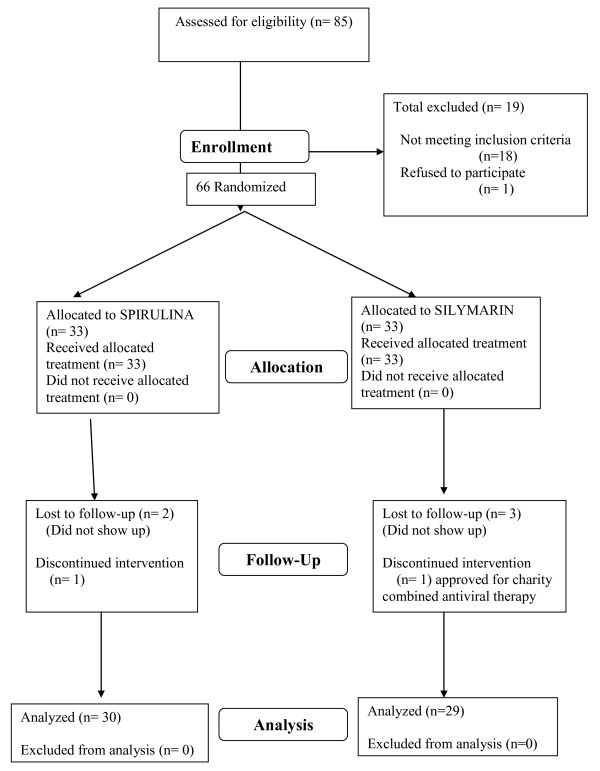
**Flowchart of patients**.

Sixty six eligible patients had been randomized to either Spirulina or Silymarin treatment groups. All patients had been followed up for a period of 6 months treatment. Four patients from Silymarin group and three patients from Spirulina group did not complete the protocol (see Flowchart in Figure 1).

The baseline characteristics were almost matched in both groups (Table 1).

**Table 1:** Baseline characteristics of analyzed patients

Characteristic	Spirulina treated Group (n = 30)	Silymarin treated group (n = 29)
Age-yr (mean ± SD)	47 ± 12	48 ± 12

Sex - M/F	21/9	18/11

Body mass index	29.8 ± 6.2	29.7 ± 6.8

Baseline HCV RNA (log_10 _IU/mL): median (Q1-Q3)	5.35 (4.84-6.07)	5.43 (4.94-6.02)

Baseline ALT: median (Q1-Q3)	76 (44-106)	75 (43-108)

Baseline Child-Pugh score: mean(SD)	7.067 (1.28)	7 (1.25)

Interferon history: (Naive/Relapser/Non-responder)	23/3/4	20/3/6

**Figure 2:**
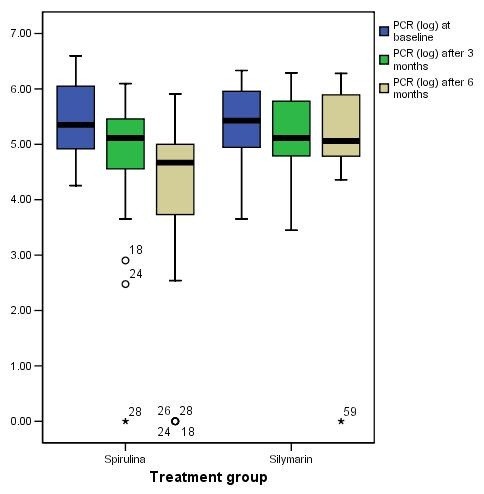
**Boxplot graph representation of log transformed virus load (Log PCR) in each treatment group**.

Among the 30 patients who had been treated with Spirulina and completed the 6 months protocol, 4 patients (13.3%) had a complete end of treatment virological response. While 2 patients (6.7%) had partial end of treatment response defined as significant decrease of virus load of at least 2-log_10 _at the end of 6 months treatment. No virological response (non-ETR) (defined as no reduction of at least 2 logs_10 _from the baseline virus load) was reported in the remaining 80% of Spirulina treated group (Table 2). While the Silymarin treated group showed complete end of treatment virological response in only one case (3.4%) and no virological response in the remaining 96.6% of the 29 patients who completed the full protocol. Though the proportion of responders in Spirulina group was greater than in the Silymarin group; the difference was not statistically significant at the end of both 6 months (p = 0.12) and 3 months treatment (p = 0.22) by Exact test (Table 2). The log transformed virus load levels at baseline, 3 months and 6 months follow up visits for each treatment group were further illustrated in a Boxplot graph in (Figure 2).

**Table 2:** End of treatment and early virological response in both groups

Treatment group		End of Treatment Response (6 months)			Total	Sig
		**non-ETR**	**p-ETR**	**c-ETR**		
	
Spirulina	Count	24	2	4	30	0.12
	
	% within Treatment group	80%	6.7%	13.3%	100%	
	
Silymarin	Count	28	0	1	29	
	
	% within Treatment group	96.6%	0%	3.4%	100%	

**Treatment group**		**Early Virological Response (3 months)**			**Total**	**Sig**

		**non-EVR**	**p-EVR**	**c-EVR**		
	
Spirulina	Count	27	2	1	30	0.22
	
	% within Treatment group	90%	6.7%	3.3%	100%	
	
Silymarin	Count	29	0	0	29	
	
	% within Treatment group	100%	0%	0%	100%	

All the 4 Spirulina treated patients who exhibited c-ETR were from those who had presented with low baseline viremia (virus load ≤ 100000 IU/mL). The lower the baseline viremia the higher was the response rate, as shown in a 3 × 3 table (Table 3) and verified by Fisher's Exact Test (value = 8.9, p = 0.028). Whereas out of those patients with baseline virus load less than 2 millions IU/mL, 6 of 27 (22.2%) in Spirulina group versus 1 of 28 (3.6%) in Silymarin group exhibited at least a partial or complete virological response at the end of 6 months treatment. The calculated Z value was 1.67 which was not significant (p = 0.09) at the predetermined "two sided" 5% level of significance, but it reached statistical significance with "one sided" Z test for proportions (p = 0.047).

**Table 3:** Relation of ETR to baseline virus load

Treatment group	Baseline viremia		ETR			Total
				
			non-ETR	p-ETR	c-ETR	
**Spirulina**	Low viremia ≤ 100000	Count	**5**	**1**	**4**	**10**
	
		% within viremia	**50%**	**10%**	**40%**	**100%**
	
	Intermediate viremia	Count	**16**	**1**	**0**	**17**
	
		% within viremia	**94.1%**	**5.9%**	**0%**	**100%**
	
	High viremia > 2 millions	Count	**3**	**0**	**0**	**3**
	
		% within viremia	**100%**	**0%**	**0%**	**100%**
	
	Total	Count	**24**	**2**	**4**	**30**
	
		% within viremia	**80%**	**6.7%**	**13.3%**	**100%**

Prior to therapy, elevated serum alanine aminotransferase (ALT) had been reported in 17 out of the 30 Spirulina assigned patients and in 15 of the 29 Silymarin group. The mean (SD) of baseline serum ALT was 74.7 (35) and 78 (33.7) respectively. At the end of 6 months therapy there was a statistically significantly greater reduction in serum ALT in Spirulina treated group than in Silymarin group by one way ANOVA (F = 8.15, P = 0.006) with mean (SD) = - 23.7 (22.3) and -6.8 (23.2) respectively.

**Figure 3:**
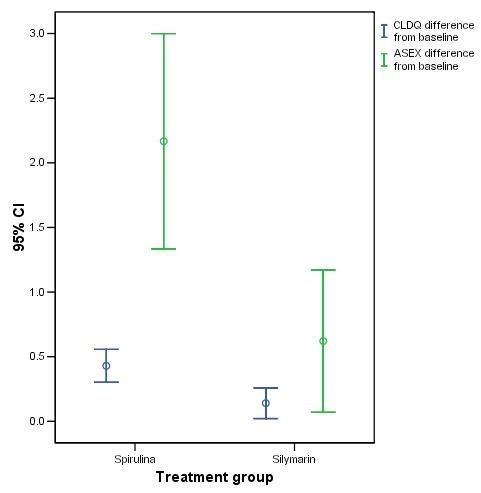
**Error-Bar graph depicting the mean ± (CI) difference from baseline in both CLDQ and ASEX scores after 6 months in each treatment group**.

Both health related quality of life scales as measured here by the Chronic Liver Disease Questionnaire (CLDQ) overall scores, and the sexual functions measured by Arizona Sexual Experience Scale (ASEX) total scores were statistically significantly improved in both treated group at the end of 6 months. See (Table 4). The mean differences in both scale scores from baseline were found to be statistically significantly greater in Spirulina treated group than the Silymarin treated group (CLDQ, F = 11.45, p = 0.001); and (ASEX, F = 9.9, p = 0.003). See (Figure 3).

**Table 4:** Mean CLDQ and ASEX scores in both groups

Treatment groups	Compared Pairs	Number	Variable name	Mean	SD	Paired t	**Sig**.
**Spirulina**	Pair 1	30	CLDQ before Rx	4.730	0.320	6.884	0.0000
				
		30	CLDQ after 6 months	4.301	0.442		
	
	Pair 2	30	ASEX before Rx	17.333	4.452	5.323	0.00001
				
		30	ASEX after 6 months	15.167	2.995		

**Silymarin**	Pair 1	29	CLDQ before Rx	4.727	0.354	2.416	0.0225
				
		29	CLDQ after 6 months	4.586	0.395		
	
	Pair 2	29	ASEX before Rx	17.241	4.265	2.306	0.0287
				
		29	ASEX after 6 months	16.621	3.959		

Multivariate analysis of covariance (MANCOVA) using the treatment group as the fixed independent factor while adding the baseline Child-Pugh score and the pre-treatment virus load as covariates showed that the treatment group (Hotelling's Trace value = 0.531, p < 0.0001) and the baseline Child-Pugh score (Hotelling's Trace value = 0.153, p = 0.021) exhibited significant effects on the vector of the two outcome variables (the improvement in CLDQ and ASEX scores), while the baseline virus load did not (p = 0.15)

It was also clear in the univariate analysis (Table 5) that the univariate effects of the corrected model and the treatment groups adjusted for both the baseline Child-Pugh score and virus load on each one of the outcome variables (CLDQ and ASEX) were statistically significant (Table 5).

**Table 5:** Univariate Analysis of Covariance Tests of Between-Subjects Effects

Source	Dependent Variable	F	**Sig**.	Partial Eta Squared
Corrected Model	CLDQ difference from baseline	5.968	.001	.246
	
	ASEX difference from baseline	7.523	.000	.291

Baseline Child-Pugh score	CLDQ difference from baseline	.033	.856	.001
	
	ASEX difference from baseline	8.314	.006	.131

Baseline virus load	CLDQ difference from baseline	3.880	.054	.066
	
	ASEX difference from baseline	.000	.997	.000

Treatment group	CLDQ difference from baseline	13.974	.000	.203
	
	ASEX difference from baseline	10.152	.002	.156

No serious adverse effects attributable to therapy were reported in both groups, apart from mild transient nausea, bloating, giddiness and headache in less than 6 patients in both groups.

## Discussion

To our knowledge, this study is the first human trial to address the effect of Spirulina platensis whole unfractionated dried extract on virus load, liver function, health related quality of life and sexual functions in patients with chronic HCV. There is only so far one article in Medline by Băicuş C, et al^.^[43] who did study the effect of one month treatment for chronic HCV patients with Spirulina on serum aminotransferases and general state with unfavorable results.

We opted to use the whole herbal extract and not any one of the fractionated bioactive molecules presuming that the whole natural multi-components as previously discussed in introduction could offer not only antiviral activity but also other immune enhancing activities that might be summated together to produce therapeutic effects on this state of chronic viral infection that evades the immune system.

Our results showed significantly greater effects of Spirulina than Silymarin on most studied parameters including the significantly greater reduction of serum ALT and the greater improvement in both disease specific health related quality of life and sexual functions scores. Though the virological response rates were not statistically significantly different between the 2 treatment groups, yet it reached the level of significance with the one sided Z test for proportions in those who presented with low or intermediate baseline viremia.

We did not note considerable changes in both virus load and serum ALT early in the first month as it seems that the beneficial effects take some times to appear as we found that improvements in most parameters were even higher after 6 months treatment than after 3 months.

We hypothesized that there must be a time needed to establish and solidify the immune mechanisms behind the activation, release and action of endogenous interferons and other interplaying cells and mediators. Even the parenterally administered high dose of interferon alpha took some months to manifest its maximal virological response, that is why we wait at least 3 months to predict the sustained virological response through the early complete or even partial virological response.

It is the main limitation of our study that we did not follow up patients for one year treatment followed by 6 months off-treatment period as the case in the protocols for the study of interferon alpha based therapy. We designed this relatively short term pilot study to answer a simple research question; is there any therapeutically feasible potential for Spirulina in chronic HCV patients, worthy to conduct a larger study with longer follow up period.

The motivation to conduct this study, "apart from the theoretically convincing background", was the unintended data coming from some of our patients who took Spirulina as nutritional supplement and reported to us marked improvement of the general well being and sexual activity. From this probing experience, as well as from the results of Danoff A, et al. [44] and Soykan A, et al. [45] who reported an association of chronic HCV with depressed sexual functions independent of depression, we opted to compare the effect of both treatments on sexual functions beside the other efficacy parameters in such patients. We assumed that improvement in sexual appetite; frequency and performance are logical indicators for the improvement in the overall wellbeing. Our results went in agreement with this assumption. Any how further large sized, randomized controlled studies with longer follow up periods are needed to confirm our results.

This study was mainly focusing to help a considerable percentage of chronic HCV patients who are facing the situation of contraindication, intolerability or non-response to the current gold standard therapy. They usually become feeling hopeless and unsecure with deterioration in their overall wellbeing, functional status and quality of life. If further studies confirm our results with reproducibility, this could be an alternative treatment in such situations if at least it can improve quality of life, physical activity and performance. We did not focus on the luxury of sustained virological response at this exploratory stage, but it will be our objective in the coming planned study.

The raised issues from the already discussed in-vitro and preclinical data about the potential immune stimulation and virus entry blocking also urged us to plan to test a new hypothesis; could the complementary therapy with Spirulina improve the response to the current gold standard antiviral therapy?. This will be tried to answer in our next study; through testing the effect of combining Spirulina with the current gold standard therapy, or the effect of offering a lead-in course for those who have high baseline virus load.

## Conclusions

From our results we can conclude that there is a therapeutically feasible potential for Spirulina platensis dried extract worthy to conduct a larger sized study with a longer follow up period to confirm these safety and efficacy encouraging results in the treatment of chronic hepatitis C infected patients.

## Competing interests

Beovita-Safe Pharma, a Joint German Egyptian Company, Katzbachstr. 29, D-10965 Berlin, had supplied the drugs and partly the costs of the laboratory tests.

## Authors' contributions

MY conceived of the study, formulated the research question, participated in its design, coordination and conduction. He took active part in all the process from patient screening to final assessment, statistical analysis and manuscript writing. AS shared MY in all the steps from screening of patients to manuscript writing and approval.

## Pre-publication history

The pre-publication history for this paper can be accessed here:

http://www.biomedcentral.com/1471-230X/12/32/prepub
